# Synchrotron Radiation Micro-CT at the Micrometer Scale for the Analysis of the Three-Dimensional Morphology of Microcracks in Human Trabecular Bone

**DOI:** 10.1371/journal.pone.0021297

**Published:** 2011-07-07

**Authors:** Aymeric Larrue, Aline Rattner, Zsolt-Andrei Peter, Cécile Olivier, Norbert Laroche, Laurence Vico, Françoise Peyrin

**Affiliations:** 1 CREATIS, Inserm U1044, CNRS 5220, INSA Lyon, Université Lyon I, Université de Lyon, Villeurbanne, France; 2 ESRF, Grenoble, France; 3 LBTO, Inserm U890, IFR143, IFRESIS, Université de Lyon, St Etienne, France; 4 Université Paris 10 – Ouest Nanterre La Défense, PST/IUT de Ville d'Avray, Département GTE, Ville d'Avray, France; National Institute of Health, United States of America

## Abstract

Bone quality is an important concept to explain bone fragility in addition to bone mass. Among bone quality factors, microdamage which appears in daily life is thought to have a marked impact on bone strength and plays a major role in the repair process. The starting point for all studies designed to further our understanding of how bone microdamage initiate or dissipate energy, or to investigate the impact of age, gender or disease, remains reliable observation and measurement of microdamage. In this study, 3D Synchrotron Radiation (SR) micro-CT at the micrometric scale was coupled to image analysis for the three-dimensional characterization of bone microdamage in human trabecular bone specimens taken from femoral heads. Specimens were imaged by 3D SR micro-CT with a voxel size of 1.4 µm. A new tailored 3D image analysis technique was developed to segment and quantify microcracks. Microcracks from human trabecular bone were observed in different tomographic sections as well as from 3D renderings. New 3D quantitative measurements on the microcrack density and morphology are reported on five specimens. The 3D microcrack density was found between 3.1 and 9.4/mm3 corresponding to a 2D density between 0.55 and 0.76 /mm2. The microcrack length and width measured in 3D on five selected microcrack ranged respectively from 164 µm to 209 µm and 100 µm to 120 µm. This is the first time that various microcracks in unloaded human trabecular bone - from the simplest linear crack to more complex cross-hatch cracks - have been examined and quantified by 3D imaging at this scale. The suspected complex morphology of microcracks is here considerably more evident than in the 2D observations. In conclusion, this technique opens new perspective for the 3D investigation of microcracks and the impact of age, disease or treatment.

## Introduction

The concept of bone quality is increasingly considered to be an important factor to explain bone fragility in addition to bone mass [1]. Bone quality refers to a number of features related to the material composition of bone and its microstructural organization. It includes many factors such as bone micro-architecture, mineralization, turnover, lacuno-canalicular system and microdamage.

Microscopic damage accumulates in bone tissue due to physiological loading and mechanical stress occurring in daily life. Microdamage has been shown to increase dramatically with age at different bone sites in humans [2], [3], [4]. A high level of microdamage is also found after a bisphosphonate treatment of osteoporosis at the same time as a reduction of bone remodeling [5], [6]. Microdamage is often associated to bone fragility fractures and is thought to have a marked impact on bone strength [7], [8].

Microdamage presents in the form of microcracks, whose size, morphology and localization are strongly related to the mechanical loading applied to bone [9], [10], [11]. Microcracks are usually classified into four types : linear, parallel, cross-hatch and diffuse [7], [12]. Initiation and propagation of microcracks have mostly been studied in cortical bone after applying *ex-vivo* mechanical loading. Four toughening mechanisms of bone tissue have been described: plastic deformation, bridging [13], creation of non-connected small linear microcracks and deflecting cracks with microstructure interfaces (cement lines) [14], [15]. In addition to dissipating energy, microdamage is also hypothesized to drive bone remodeling by sending stimuli to osteocytes and plays a major role in the repair process[16]. In particular, a decline in osteocyte lacunar density has been shown to be associated with an accumulation of microcracks [17].

Although many studies have been conducted in animal models, recent data have been reported on human bone specimens [9], [13], [18], [19]. Less attention has been devoted to microcracks in trabecular bone [2], [12] in the past, but this topic is now becoming the subject of growing interest [10]. While the porosity and the anisotropy of trabecular bone tissue make it difficult to work with, studies on trabecular bone allow the analysis of microarchitecture simultaneously with microdamaging [2], [16], [20], [21].

The most widespread technique for *in vitro* investigation of microdamage consists in observing thin slices of bone by microscopy after staining, which requires histological sectioning of bone specimens [22], [23]. Scanning Electron Microscopy has a much smaller field of view and is often used as a complement to observe a small number of microcracks [14] at very high resolution.

Microcracks are generally described as thin planar ellipsoids whose thickness is of the order of the micrometer [16]. They are usually counted and measured manually, which is likely to be associated with some variability. In addition, two-dimensional (2D) sections provide incomplete information about the complex three-dimensional morphology and size of microcracks. Also, because microcracks can be relatively scarce, the measure of their density in cross-sections can be very sensitive to sampling effects [24]. Although 2D measurements can be extrapolated to three-dimensional (3D) measurements by using statistical models [24], [25], such models require prior knowledge and may not necessarily be valid for an individual microcrack. Ideally, bone microcracks should therefore be observed, analyzed and measured in three dimensions with isotropic and sufficiently high spatial resolution.

3D observations of microcracks in cortical bone were obtained by reconstruction of microscopic images after serial sectioning [26], [27]. Confocal microscopy can also produce micrometric 3D images of microdamage [28], [29], [30] but with an anisotropic resolution and a small depth (typically 200 µm). Finally, contrast agents are being developed for 3D observations with standard micro-CT devices [31], [32], [33]. Even if this technique is able to detect the presence, spatial location, and accumulation of microdamage, the spatial resolution of these images and the capacity of contrast agents to specifically bind to microcracks are still not sufficient to provide relevant 3D data on microcrack morphology.

Synchrotron Radiation Micro-Computed Tomography (SR micro-CT) possesses significant advantages over standard micro-CT. A synchrotron source provides a high-flux, high-intensity and monochromatic X-ray beam, allowing acquisition of quantitative high-resolution 3D images with a high signal-to-noise ratio [34]. SR micro-CT has already been used to study trabecular microarchitecture, remodeling and local mineralization [35]. More recently, this technique was used to study the propagation of long linear cracks or microcracks when applying mechanical loading [19], [36], [37], [38]. Only the first study [36] concerned trabecular bone but with bovine specimens and at a resolution relatively low (7 µm) for microdamage assessment.

In this paper, we present a new 3D imaging method based on SR micro-CT to analyze quasi-physiological microcracks in human trabecular bone at the micrometric scale. The precision of SR micro-CT and a tailored image processing technique allow detecting, observing and analyzing the morphology of microcracks at the considered scale.

## Results

### 3D micro-CT imaging

**Figure 1:**
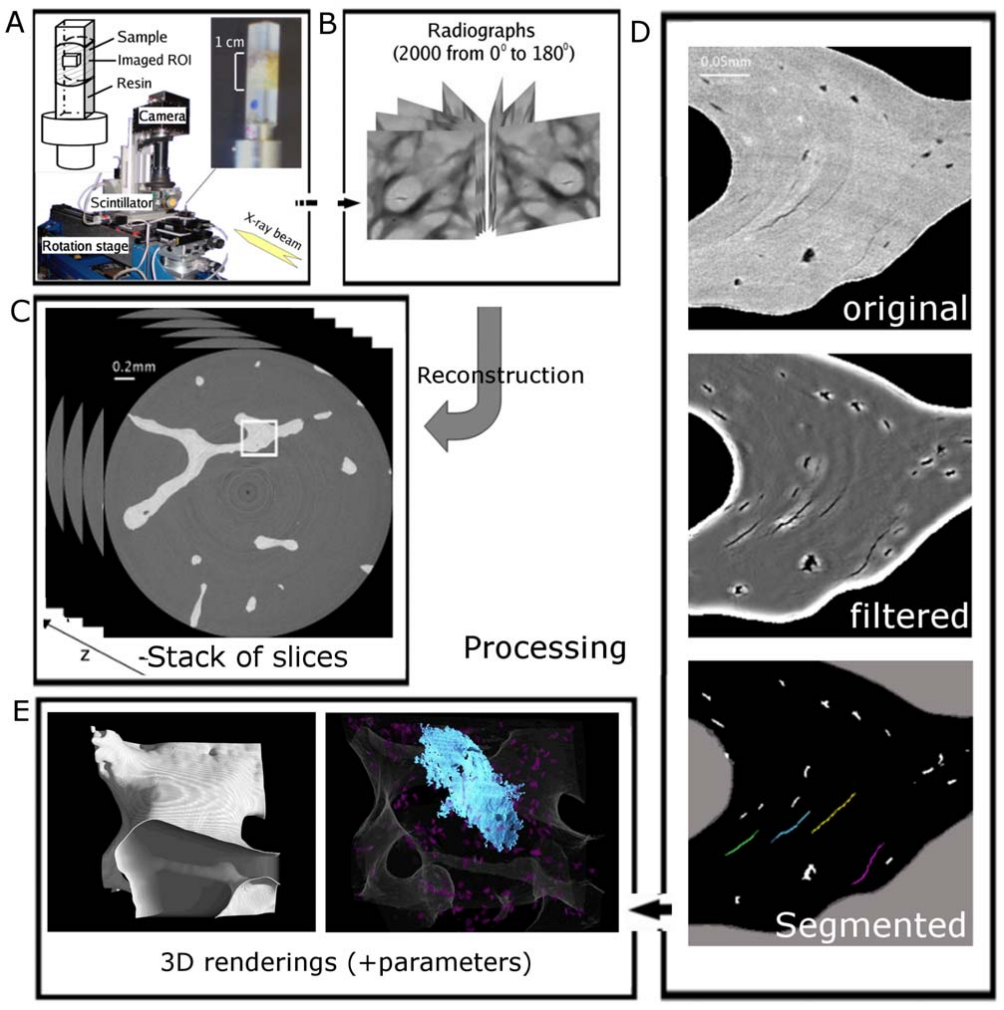
Diagram of the imaging process. A: specimen (photograph and diagram) and specimen stage. Specimens are 5×5×10 mm and the imaged field of view is 2.8×2.8×2.2 mm. B: 2000 radiographs of the specimen are taken from evenly distributed angles from 0° to 180°. C : after tomographic reconstruction, the 3D image is obtained, composed of a stack of slices. D : 256×256 ROI of a reconstructed grey-level image(top), customized non-linear filtering (middle), and segmentation (bottom, microcracks in color, lacunae in white). E: 3D renderings can then be produced to observe and measure microarchitecture or microcracks.

Fig. 1A–B illustrates image acquisition of a specimen of trabecular femoral bone on the SR micro-CT setup developed on beamline ID19 at the ESRF (European Synchrotron Radiation Facility), Grenoble, France. Two thousand radiographs of the each specimen were acquired at rotation steps of 0.09° to cover a total angular range of 180°. Reconstruction by a 3D Filtered Backprojection algorithm produces a stack of slices which forms the 3D image of the specimen (Fig. 1C). In the region of interest shown in Fig. 1D (top), differences in gray levels reflect different degrees of mineralization due to bone remodeling. Osteocyte lacunae are clearly visible. Four microcracks can be seen but are weakly contrasted mainly because of partial volume effect, noise corruption and ring artifacts due to nonlinear detection.

### Observation of microcracks in 2D micro-CT slices

**Figure 2:**
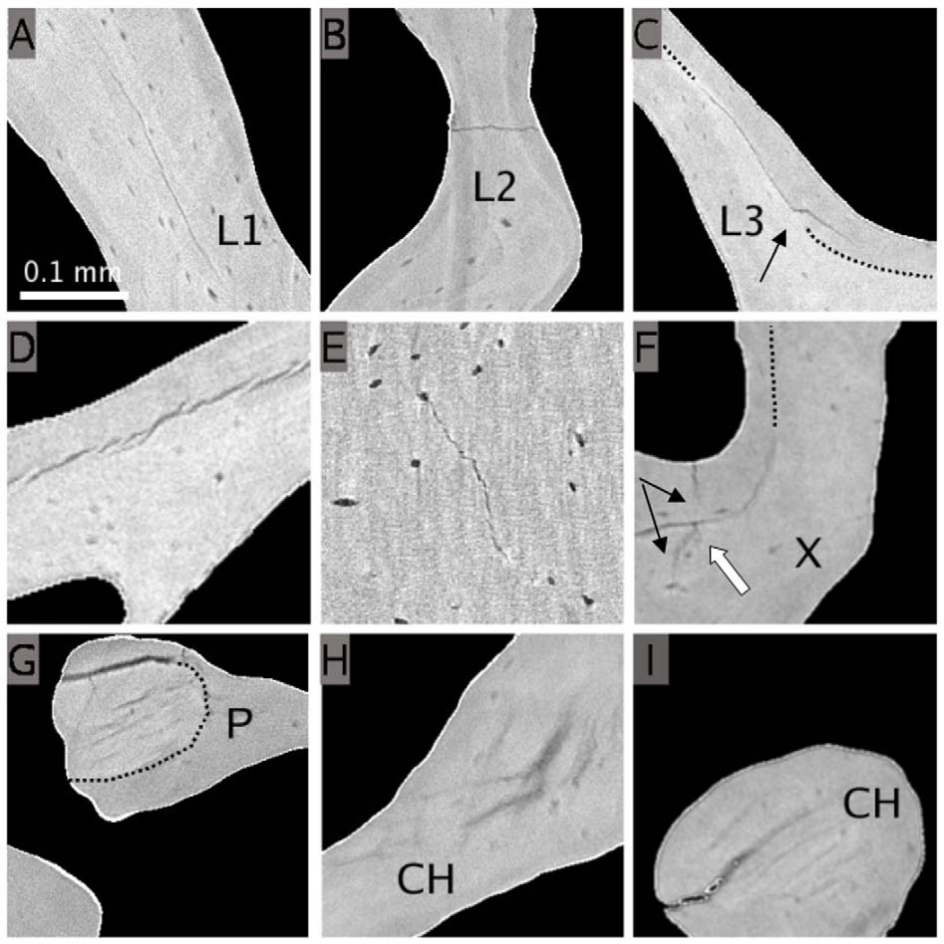
Illustration of various types of microcracks from 2D slices of SR micro-CT images. A : long linear microcrack, parallel to the trabecular surface(L1). B: microcrack dividing a trabecula(L2). C : microcrack deflected (black arrow) by the interface between two areas of different mineralization (dots) (L3). D : discrete cracking or bridging. E : tortuous microcrack crossing a lacuna. F : crack (X) perpendicular to the trabecular surface and appearing to be split (black arrow) and crossing a crack (white arrow) driven by a cement line (black dots). G : parallel microcracks contained in a uniformly mineralized area (P). H–I : cross-hatch cracks (x–y plane and x–z plane views) which appear to be parallel in the x–z plane (CH).

The qualitative examination of 2D micro-CT slices allowed the observation of various morphologies of microcracks. Fig. 2 shows a series of observations revealing interesting features. Fig. 2A presents a large linear microcrack parallel to the bone surface (denoted L1 for “linear n^o^1”), while Fig. 2B shows a microcrack perpendicular to the surface (denoted L2 for “linear n^o^2”) that appears to split the trabecula. The microcrack in Fig. 2C (denoted L3 for “linear n^o^3”) arises in a poorly mineralized region, is deflected (black arrow) and finally follows a cement line (lower intensity in the upper part of the crack). This type of behavior, known as microcrack deflection, has already been described as a way to prevent trabecular breaking [14]. In Fig. 2D, numerous discrete small cracks following the cement line can be seen, illustrating another mechanism to dissipate deformation energy. Fig. 2E exhibits a case in which crack propagation started (or stopped) in a lacuna. The microdamage area shown in Fig 2F (denoted X for “cross”) includes a linear crack parallel to the surface and following a cement line (white arrow) and another microcrack (that appears to have split, black arrows) perpendicular to the trabecula. Parallel linear cracks (denoted P for “parallel”) can be observed in Fig. 2G where they appear to be confined to a homogeneously mineralized zone. 3D micro-CT images enable to visualize cracks in orthogonal planes. Fig. 2H and 2I show the same microcracks on the x–y and x–z planes. Although these microcracks (denoted CH for “cross-hatch”) are clearly cross-hatch (Fig. 2H), they appear to be parallel microcracks when observed on a 2D single orthogonal slice like in Fig. 2I and could be easily misinterpreted.

### Control of microcracks images

The existence of microcracks observed in micro-CT images was controlled by epifluorescence microscopy. On that purpose, a slightly different protocol was applied (cf. Materials and Methods). 400 µm-thick sections were first observed by epifluorescence microscopy at different magnification, and then imaged by SR micro-CT. In epifluorescence microscopy images, a fluorescing chelating agent (calcein) makes the microcracks visible.

**Figure 3:**
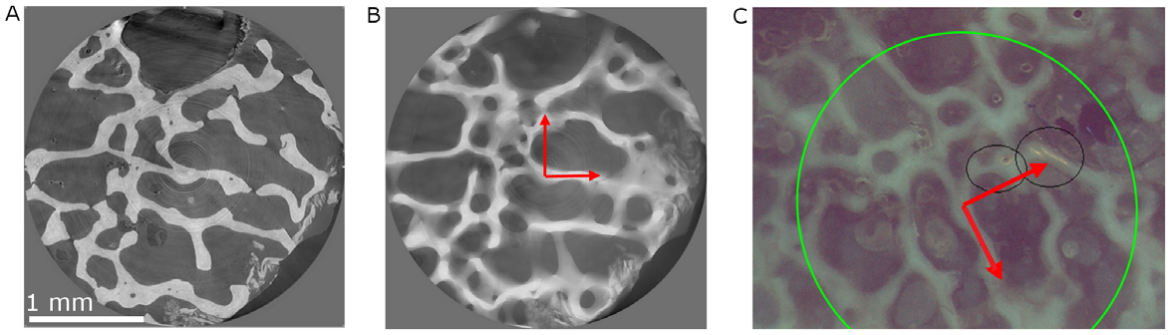
Identification of the same region of the specimen in both SR micro-CT images and epifluorescence microscopy images at low magnification. A : Original SR micro-CT slice. B : Image computed by averaging the grey levels along the z-axis of the stack. C: Epifluorescence microscopy image showing the same region (green circle) but with a different orientation (cf. red arrows). The black circles contain a microcrack and a pore

A slice of a SR micro-CT image (cf. Fig. 3A) corresponds to a micrometric section whereas the microscopy image is produced by a light passing through the whole specimen (cf. Fig. 3C). As a consequence, the identification of the same structures in both images is not straightforward. By averaging the grey level values along the z-axis (cf. Fig. 3B), the effect of thickness was simulated on micro-CT images. This allowed the identification of the regions of interest imaged with micro-CT in the low magnification microscopic images. Then, individual microcracks found in micro-CT images were looked for in the corresponding high magnification microscopic images. In most cases, the same microcracks could be observed in both images.

**Figure 4:**
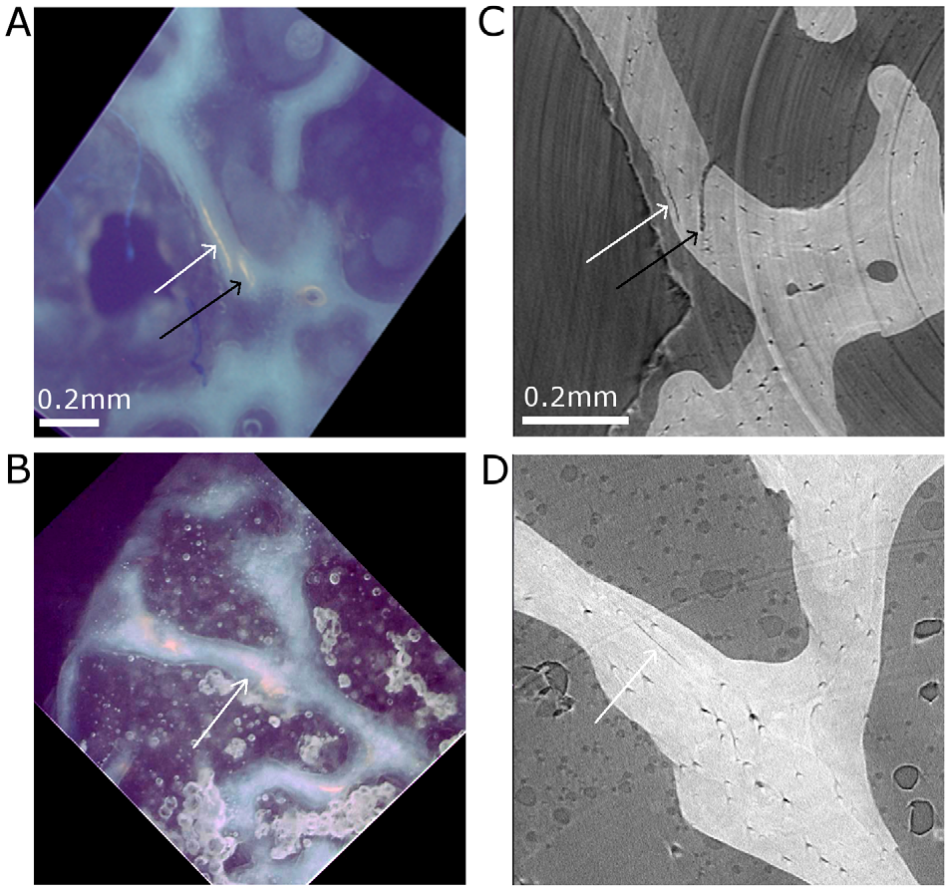
Identification of the same microcracks in images from epifluorescence microscopy at high magnification and SR-micro-CT. A–B: image from epifluorescence microscopy showing microcracks (white arrows) and an artifact (black arrow). C–D: Corresponding image by SR micro-CT. The orientation of the epifluorescence microscopy images had to be corrected.

As an illustration, Fig. 4 shows the same microcracks observed with the two modalities. Fig. 4A–B show a microscopic observation of microcracks (white arrows) at high magnification and Fig. 4C–D show the same region of interest and the same microcracks (white arrows) in the corresponding slice in the 3D SR micro-CT image. Although the imaged region is not exactly the same and the scale is different, trabeculae and marrow spaces can be readily identified by their shape. Note that the microcracks can look thicker and longer in the epifluorescent image which can be due to the thickness of the section. Whereas the higher spatial resolution available in SR micro-CT image reveals that the second porosity in Fig. 4C (cf. black arrows) is not a microcrack but a larger porosity, the epifluorescent image (Fig. 4A) shows a fuzzy spot.

### Segmentation of micro-cracks

**Figure 5:**
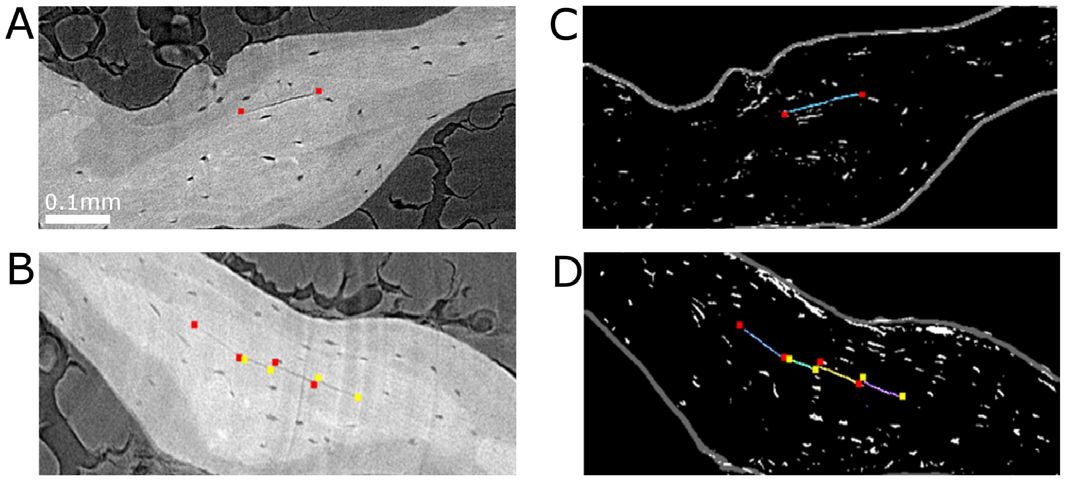
Evaluation of the segmentation method. A–B: Original SR micro-CT image showing microcracks. Their extremities have been marked with a yellow or red dot. C–D: Segmented images with the extremities of the microcracks reported. The microcracks are well detected and segmented.

The observation of micro-cracks in 3D - namely the synthesis of 3D renderings - requires their segmentation, i.e. the definition of their boundaries in the image. Since each 3D image is composed of 1500 slices of 2048×2048 pixels, the manual segmentation of micro-cracks would be tedious and time-consuming. A procedure was thus developed to automatically identify microcracks (cf. Material and Methods). Fig. 1D illustrates the main steps of the methods based on an original filtering, followed by hysteresis segmentation and a labeling separating microcracks from potential artifacts and lacunae. The reliability of the proposed method was tested by comparing the manual and automatic segmentation of 30 microcracks. Fig. 5 illustrates the agreement between the automatic segmentation (micro-cracks in color) and the manual segmentation. Each extremity of the microcracks has been defined in the original image (Fig. 5A–B) and reported as yellow or red dots in the segmented image (Fig. 5C–D).

### 3D renderings of microcracks

**Figure 6:**
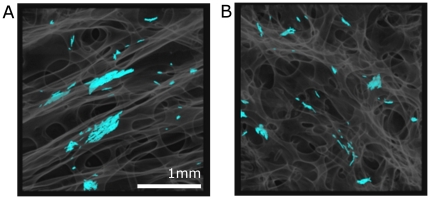
3D renderings of microcracks (in blue) in trabecular bone specimens 4 and 2. The bone surface is shown in transparent white, revealing the microarchitecture (view from the top the specimen).

**Figure 7:**
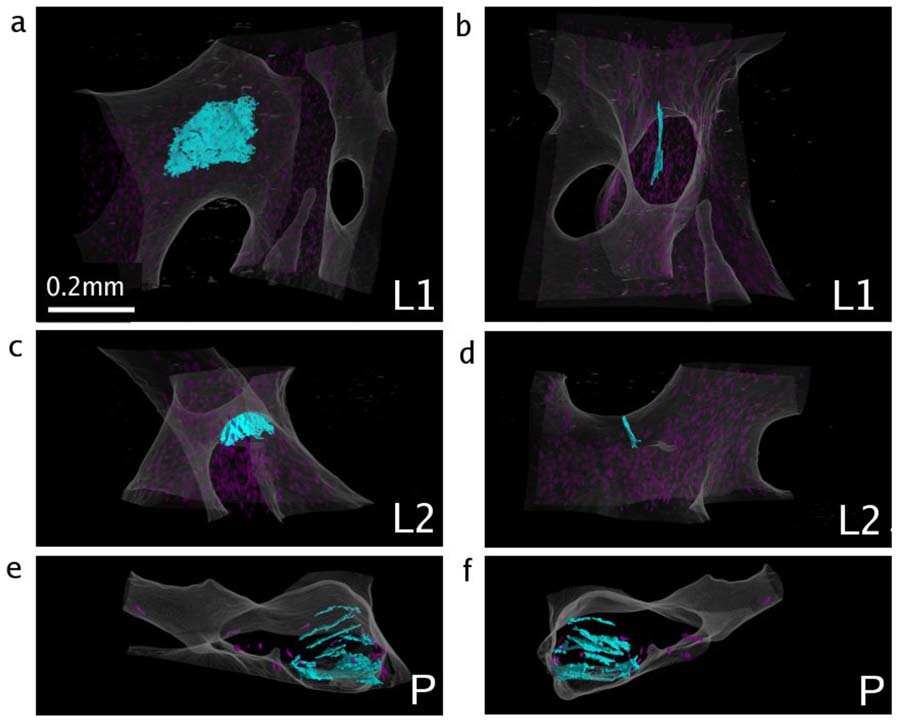
3D renderings of various types of microdamage. A–B: large and thin linear microcrack (L1), parallel to the surface (front and side view, 512×512×512 ROI). C–D : crack (L2) perpendicular to the surface and matching the shape of the trabecula (front and side view, (512×512×256 ROI). E–F : Parallel cracks(P) (256×256×128 ROI).

The application of this automatic segmentation method on the reconstructed images allowed 3D observations of various types of microcracks, as illustrated on Fig. 6, 7, 8 *(see also Movie S1 in the Supplementary Information)*. Microcracks are displayed in blue under different angles of view, lacunae in translucent purple and bone envelopes in translucent white.

**Figure 8:**
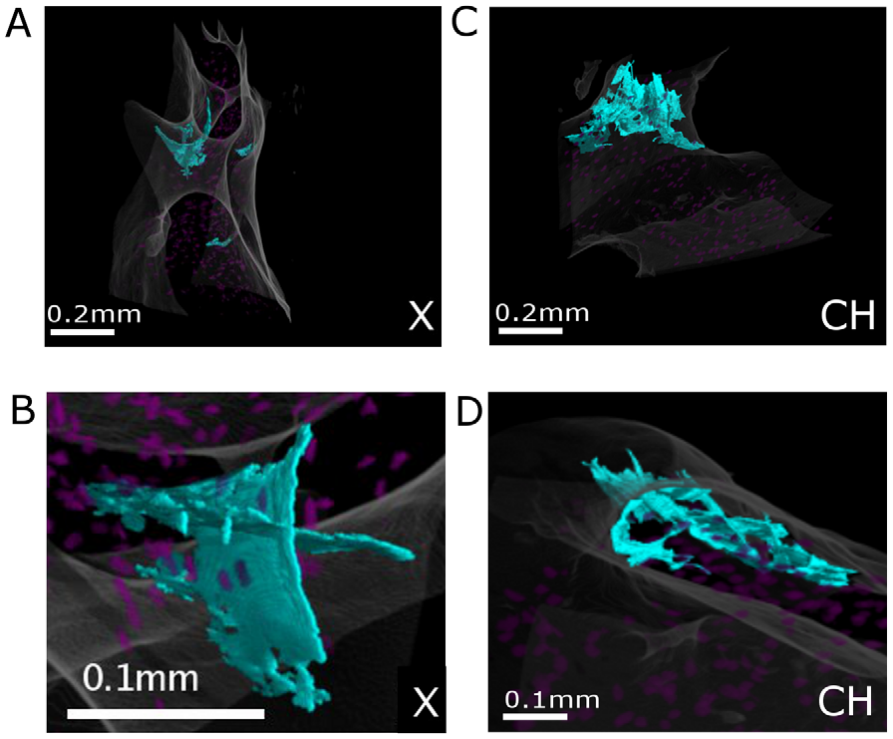
3D renderings of crossed cracks (X), and cross-hatched microcracks (CH). (512×256×256 ROIs). A: global view of X showing it is actually composed of two twisted cracks, B : zoom (from different angle), C: global view of CH showing it spreads all over the trabecula, D: zoom showing it is made of two series of parallel linear microcracks.

Figure 6 presents top views of two segmented images showing the global repartition of microcracks within the entire specimen. Differences can be found between the two specimens. The trabecular micro-architecture of the first specimen (Fig. 6 A) is strongly oriented while the second (Fig. 6 B) seems more isotropic. In addition, the first specimen contains larger linear microcracks than the second. In both cases, the microcracks appear to be quite homogeneously distributed within the specimens.

Fig. 7A–B present 3D renderings of L1, which appears to be very thin, parallel to the trabecular surface, with a trapezoidal rather than ellipsoidal shape. Closer inspection shows that this crack is locally interrupted, which can be attributed to uncracked bone ligaments. Fig. 7 C–D reveal that L2 only partially traverses the trabecula, matches its shape, and is orthogonal to its surface. The parallel microcracks (P) are displayed in Fig. 7E–F. In Fig. 8, the complex microcrack X (A:global view, B:zoom) appears to be the union of two twisted planes crossing each other. Fig. 8 C–D show that the cross-hatched cracks actually spread within the whole trabecula, forming a large damaged area (cf Fig. 8. C). In the zoom Fig. 8D, they appear clearly as two series of parallel linear microcracks crossing each other.

### 3D quantitative data on microcracks

**Figure 9:**
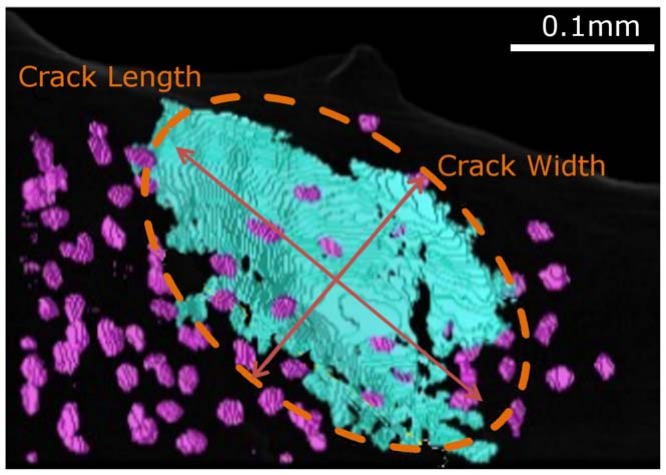
3D rendering of a microcrack with its best-fitting ellipsoid. The first two axis of the ellipsoid can be identified as the length and width of the microcrack.

In addition to providing 3D observations, segmentation allows quantitative measurements on microcracks. The quantification of microcracks and lacunae was performed from the labeled image. The microcrack volume (Cr.V) was obtained directly by counting the number of voxels while microcrack length and width were computed as the axis of the best-fitting ellipsoid (cf. Fig. 9). The microcrack thickness (Cr.Th) was then estimated as the mean value of the microcracks voxels in the 3D direct thickness map and the microcrack surface area (Cr.S) was evaluated as the microcrack volume divided by its thickness.

Table 1 reports the 3D quantitative measurements on the selected microcracks presented in Fig. 2 and Fig. 6, 7, 8 (length: Cr.Le, width: Cr.W, thickness: Cr.Th, volume: Cr.V, and surface: Cr.S; average values for P). For each of them, the thickness of the trabecula containing the microcrack (Tb.Th.lo) and its nature (linear and/or deflected) are also given. Global quantitative data of the five specimens are given Table 2. The number and density of microcracks (Cr.N and Cr.Dn) found in each specimen, their mean dimensions (length: Cr.Le mean, width: Cr.W.mean, thickness: Cr.Th.mean) and the respective standard deviations are reported in Table 2. Bone volume fraction (BV/TV), structure model index (SMI) and lacunar density (La.Dn) are also provided. In addition, Table 2 presents the results of the simulation of 2D measures on the specimens (cf. Materials and Methods): bone volume fraction (BV/TV 2D), microcracks mean length (Cr.Le2D mean), number (Cr.N.2D), and density (Cr,Dn.2D).

**Table 1:** 3D quantitative data measured on the selected microcracks.

microcrack		L1	L2	P	X	CH	L3
sample		1	2	3	2	4	5
Cr.Le	(µm)	341	149	88	269	492	300
Cr.W	(µm)	201	78	48	132	307	190
Cr.Th	(µm)	3.11	2.91	4.73	3.11	4.73	2.91
Cr.V	x10^6^ (mm^3^)	89.1	14.85	9.8	60.96	850.65	80.73
Cr.S	x10^3^ (mm^2^)	28.64	5.10	2.07	19.60	179.84	27.74
Tb.Th.lo	(µm)	308	195	232	276	231	168
Trabecular Shape		Plate	Junction	Rod	Junction	Plate	Rod
Linear		yes	yes	yes	no	no	yes
Deflected		no	no	yes	-	-	yes

*Cr.Le: length , Cr.W :width , Cr.Th thickness, Cr.V volume,Cr.S surface area,*

*Tb.Th.lo: local thickness of the trabecula.*

**Table 2:** Microarchitecture and microdamage measurement on the five specimens.

Sample		1	2	3	4	5
BV/TV		0.22	0.19	0.16	0.20	0.12
BV/TV 2D		0,20	0,19	0,20	0,16	0,10
SMI		1.41	1.73	1.62	2.20	2.07
La.Dn /10^3^	(#.mm-3)	15.68	14.39	12.44	12,34	8.01
Cr.N	(#)	14	14	18	28	7
Cr.N 2D	(#)	10	7	8	19	4
Cr.Dn	(#.mm-3)	3,42	4,1	4,91	9,44	3,11
Cr.Dn 2D	(#.mm-2)	0.76	0.55	0.60	1.83	0.58
Cr.Le mean	(µm)	206,57	182,92	204,94	208,84	164,14
Cr.Le std	(µm)	124,43	57,5	140,65	152,01	90,92
Cr.W mean	(µm)	111	99,84	119,77	117,57	95,57
Cr.W std	(µm)	71,19	36,17	91,29	87,43	66,73
Cr.Le 2D mean	(µm)	220.5	130.8	225.23	238.15	143.15
Cr.Le 2D std	(µm)	101.88	51.20	135.36	134.03	19.45

*3D quantitative data : BVTV :: bone volume fraction, SMI : Structure Model Index,*

*La.Dn : lacunar density, Cr.N : crack number, Cr.Dn : crack density, Cr.Le mean : mean crack length, Cr.W mean : mean crack width.*

*Results* *of the 2D approach simulation: BVTV 2D : bone fraction, Cr.N 2D: crack number, Cr.Dn 2D : crack density, Cr.Le 2D mean : mean crack length.*

The mean length and width of microcracks ranged respectively from 164 µm to 209 µm and 100 µm to 120 µm, while the respective standard deviations ranged from 57 µm to 152 µm and 36 µm to 91 µm. This reflects a high level of variability of microcracks dimensions. Microcrack density was in the range 0.6.mm^−3^ except an outlier at 1.83.mm^−3^. The situation was the same for lacunar density with four values ranging from 12,000 mm^−3^ to 15,000 mm^−3^ and the fifth at 8,000 mm^−3^.

## Discussion

This study reported a new technique based on SR micro-CT for the three dimensional analysis of microcracks in human trabecular bone. SR micro-CT takes advantage of the exceptional properties of X-ray beams extracted from synchrotron radiation to acquire 3D images of specimens at very high resolution. To the best of our knowledge, this is the first time that various types of microcracks - from the simplest linear crack to more complex cross-hatch cracks - have been examined and quantified by 3D imaging at this scale and in these conditions. In this work, human trabecular bone taken at a physiologically loaded site without application of additional mechanical loading was used. Though it was a major source of difficulty, this strengthens the originality of the study and widens the range of application of the proposed method.

The new technique described here allowed observing microcracks sections under different orientations as well as in the 3D space. The first striking conclusion is that the morphology of microcracks appears to be far more complex than suggested by 2D observations. The unavoidable sampling in 2D imaging makes it impossible to render complex microcrack shapes such as twisting or crossing. In addition, 3D imaging of trabecular bone allows to inspect simultaneously the topology of the bone trabecula, that of the microcrack and their relative positions and orientations.

Imaging microcracks requires a high spatial resolution at the micrometer scale to capture such tiny porosities. At the same time, since trabecular bone has a high porosity, a sufficient field of view has to be imaged to get a representative bone tissue volume. These two requirements are contradictory since spatial resolution decreases when the field of view increases. With a voxel size of 1.4 µm and 2 scans per specimen, a total volume of ∼30 mm^3^ was imaged. The use of a voxel size of 0.7 µm would have been technically possible and would have facilitated the image analysis task but it would also have reduced by a factor 8 the total volume of analysis (∼4 mm^3^). Therefore, a voxel size of 1.4 µm was found to be a good compromise between the spatial resolution in the image and the available field of view.

A current problem when imaging microcracks is to avoid artifacts due to cutting. In isolated 6 mm diameter trabecular bone specimens, in which peripheral trabeculae lose their original load-bearing capacities, Un et al estimated [39] that a 0.2–0.6 mm wide (depending on mean trabecular separation) external ring has to be deleted from the analysis. Davies et al [40] showed that even if the preparation of the specimens contains some aggressive steps, the order in which those steps occur and the fact that each step extracts the central core of the preceding material enables to limit dramatically the creation of artifactual microcracks in the central region. Considering these findings, we only imaged out the central core of the specimen, thus excluding microdamage from the sides of the machined specimen. The qualitative observations of the SR micro-CT images confirmed that there were not more or bigger microcracks at the periphery of our images, as can be seen in Fig. 6. However, we cannot exclude other potential artifactual damage occurring during sample preparation such as dehydration, although this process was found to induce an increased tissue modulus [41].

Fluorescent microscopy was used to control the match between microcracks observable in our images and microcracks present in the specimens. Since this technique does not have the same requirements in terms of specimen size and preparation, we designed a specific protocol to image the same specimen with micro-CT and fluorescent microscopy. We also assumed that two observations of microcracks of similar length and morphology, at approximately the same position in trabeculae of the same shape can be considered as observations of the same microcracks. The results show that although the images had different characteristics, the same microcracks could be observed with both modalities. We acknowledge that the quality and the resolution of the microscopic images were not optimal but microcracks could still be identified. In addition, the careful observations of microcracks on different slices as presented on Fig. 2 has shown typical characteristics already described from 2D histological observations, which constitutes another reason to be confident concerning the nature of the observed microcracks.

A totally new image processing technique was developed to automatically segment and extract quantitative measurements of individual microcracks. Note that with the chosen voxel size, the smallest section of a microcrack is hardly larger than one voxel. When adding the contribution of noise and ring artifacts, the segmentation appears to be much challenging. To this aim, a new segmentation method based on adapted non linear filtering was proposed. Although a systematic comparison between manual and automatic segmentation was not possible, due to the large data sets, the evaluation made on randomly selected images showed a good agreement between the two methods.

New 3D parameters were defined for the quantification of microcracks in the 3D images. The total length and width of microcracks and lacunae were estimated as the sizes of the best fitting ellipsoid. Their calculation was implemented by using a 3D moment based approach. Although the actual microcrack shape may be significantly different from an ellipsoid, this method provides a good evaluation the global crack extent. Typical microcrack dimensions are in agreement with the only comparable published 3D data [26] with a length ranging between 88 µm and 492 µm and a width between 48 µm and 307 µm. No more precise comparison can be performed, as reference data [26] are limited to linear microcracks in compact bone. To our knowledge, no published data for 3D microcrack density in human bone are currently available. Values for lacunar density (La.Dn) are also coherent with previous works which report a density of roughly 13,000 mm^−3^ [42]. In order to compare our results to 2D measurements reported in the literature, we also computed 2D equivalent measurements. The estimated 2D equivalent microcrack densities revealed values between 0.55 and 1.83 mm^−2^, i.e. in the same order of magnitude as that reported in the literature [2], [3], [7], [20].

It should be noticed that the analysis of 3D images eliminates ambiguities regarding the interpretation of a 3D structure from sections only. Microcracks are very anisotropic structures and measuring them from 2D histological slices is likely to be sensitive to the arbitrary direction of cutting. As the density of microcracks is usually low and microcracks may not be evenly distributed within the specimens, considering a limited number of sections to characterize a whole specimen might produce biased measures [24]. The comparison between our 3D and simulated 2D measures shows similar trends but raises some mismatches. For instance, the microcrack density can be overestimated in 2D if the specimen includes large microcracks that are counted in several sections, and underestimated when small microcracks are missed by the studied sections (see specimens 2 and 3. For specimens 5 and 2, the 2D microcracks mean length is between the mean length and width measured in 3D. However, in specimens 1, 3 and 4, it is even higher than both 3D measures, revealing the missed cracks were the smallest. The 7 µm thickness of virtual sections used for 2D analysis was chosen in order to take into account the focus effect and the limited depth of penetration of light in thick sections. The aim was not to reproduce exactly the condition of microscopic observations, but to emphasize the differences between 3D and 2D approaches. These results highlight the high dependence of 2D measurements on the size, shape and orientation of the microcracks. The high variability of the microcracks morphology observed in 3D demonstrates the limited relevance of making any model assumption. In the same way that the superiority of direct 3D parameters compared to 2D model-based parameters is now well admitted for the quantification of trabecular bone, the same improvement can be expected for the quantification of microcracks.

We acknowledge that the technique has some limitations. First, unlike in histology, microcracks could not be labeled by a contrast agent. As a consequence, the presence of artifactual microcrack cannot be totally excluded. The development and study of contrast agents for labeling microcracks in micro-CT images is a new and active field of research [31], [32], [33]. Recently, new contrast agents were shown to allow detecting and monitoring the accumulation of microdamage in micro-CT images at 10 µm [31]. However, in this study staining was performed after the specimen preparation and loading. Hence, it does not exclude artifactual microcracks but compensate the lack of spatial resolution of standard micro-CT. In order to deal with the issue of the creation of artifactual microcracks, a site matched comparison between microcrack analysis from SR micro CT and histology is planned in further work. A second concern that could be raised is the area of analyzed bone tissue. Indeed, our 3D images have a sectional area of 2.8×2.8 mm^2^, whereas microcracks are generally observed in histological slices of larger diameter (i.e. 7.5 mm in [18]). Nevertheless, conversely to histology, the analyzed region is not an area but a small volume of about 2.8×2.8×3.7 mm^3^, i.e. ∼30 mm^3^. As a comparison, for each specimen analyzed in [18], three 100 µm-thick histological slices spaced by 300 µm were prepared and analyzed, leading to a total specimen volume of ∼39 mm^3^ from which ∼26 mm^3^ is left unobserved. Thus volumes remain in the same range whilst the geometry of the bone region analyzed by this technique differs from that in histology. Preliminary unpublished results on a larger data set indicated that the intra specimen variability was significantly smaller than the inter specimen variability. Increasing the size of the images is essentially a technological issue that will be solved in the next few years. New 4096×4096 detectors are currently appearing in the market and will lead to an increasing of the imaged volume by a factor 8. Finally, further limitations are the obviously limited accessibility of SR micro-CT and the time required to acquire and process the images. However, it should be emphasized that the preparation of specimens for SR micro-CT is less time-consuming than for histology since it does not require thin slice grinding.

In summary, the high spatial resolution of SR micro-CT allowed 3D imaging of microcracks in their environment. This approach allows a global analysis of bone properties simultaneously to a detailed characterization of bone microdamage. The possible misinterpretation of the analysis through two-dimensional approaches was highlighted with several examples. 3D imaging also reveals the high complexity of microcrack morphology. For instance, they can appear as a combination of linear microcracks rather than a simple linear microcrack. Thus the possibility of observing microcracks in 3D can shed new light in the classification of microdamage. An image processing technique was developed to automatically extract quantitative measurements from the 3D images, which is particularly important due to the large data set to process. Statistical data on microcracks in various conditions can therefore be readily obtained.

We think that this new technique will bring further insights on microcracks and trabecular microfracture, on relationship between microcracks and local surrounding trabecular bone including osteocyte lacunae, trabecular microarchitecture and degree of mineralization. Furthermore, coupling this 3D imaging technique with finite-element analysis could be a new step in the understanding of bone microdamage formation and biomechanical consequence. The knowledge of the evolution of microdamage in various types of bone tissue (cortical, trabecular, lamellar, plexiform, woven….) with age, disease, treatment and mechanical loading might greatly benefit from this new technique.

## Materials and Methods

### Sample Preparation

Femoral head trabecular bone compartments were obtained from patients undergoing total hip replacement for coxarthrosis. This project has been approved by the institutional ethic committee CPP (Comité de protection des Personnes Sud-Est I, France n°2010-167, President Philippe Rusch). In absence of personal consent, specimens were collected after consideration and approval of the research ethics committee, according to article 25 of the WMA Declaration of Helsinki (Ethical Principles for Medical Research Involving Human Subjects) and article L 1211-2 of laws “Huriet-Sérusclat” n° 2004-800 and n° 2004-806. The study population consisted of male and female subjects between the ages of 77 and 80 years with no history or evidence of genetic disease or malignancy.

20 mm thick slices of femoral heads were cut in the transverse plane by surgeons immediately after surgery. The requirements for optimal specimen preparation as described by Davies et al [40] were scrupulously respected. Cylinders, 10 mm in diameter, were drilled with a diamond trephine from the 20 mm thick slices and cut to a height of 5 mm using a Leica SP1600 Saw microtome. Throughout the cutting procedure, the bone was irrigated with sterile 0.9% sodium chloride at 4°C to limit heat-generated damage, remove bone chips, and prevent drying. Bone cores were then embedded in methylmethacrylate. Finally, pseudo-parallelepiped specimens (5×5×10 mm) taken from the central part of the cylinders (see Fig. 1A) were prepared by grinding ( ESCIL ESC200GT) for SR micro-CT imaging.

Five specimens were also prepared for epifluorescence microscopy used as a control method. On that purpose, five cylindrical specimens (h: 10 mm, 5 mm diameter) were bulk-stained by immersion in a calcein solution (0.5 µM in 80 % ethanol) and put into a vacuum chamber for 12 hours. For each of them, four 400 µm thick sections were cut with the Leica SP1600 Saw microtome.

### 3D Micro-CT Imaging

SR micro-CT was performed on beamline ID19 at the ESRF (European Synchrotron Radiation Facility), Grenoble, France, as previously described [43].

Specimens were glued on stands adapted to the rotation stage and placed as close as possible to the camera in order to limit phase contrast [44]. Crystal monochromators were used to sharply select a single X-ray energy, set to 23 keV. The X-ray beam transmitted through the specimen was acquired on a detector including a YaG scintillator screen, an optical lens and a 2048×2048 CCD FReLoN detector. A 2048×1400 ROI on the detector was used due to the limited beam height at this energy. The pixel size was set to 1.4 µm, which provides a field of view in the specimen of 2.8×2.8×1.96 mm^3^. For each specimen, two adjacent scans were acquired in the vertical direction with an overlap of 280 µm. Finally, by merging these two data sets, a total reconstructed volume representing 2.8×2.8×3.7 mm^3^ was obtained for each specimen.

Only the cores of the specimens have been imaged, thus excluding microdamage from the sides of the machined specimen. For each scan, 2000 radiographs were taken at different angles evenly distributed between 0 and 180 degrees (cf. Fig. 1B). Finally, the Filtered Back Projection algorithm was applied to obtain a reconstructed 3D volume, i.e. a stack of 1400 slices of 2048×2048. Note that since the voxel is isotropic, the slice thickness is equal to the pixel size, i.e. 1.4 µm.

#### Protocol for micro-CT imaging and epifluorescence microscopy

A specific protocol was designed to observe the same specimen by micro-CT imaging and epifluorescence microscopy. In this case, the 400 µm-thick sections prepared as described above were first observed by epifluorescence microscopy (wavelength: 546 nm; microscope: DMRB, Leitz) at different magnification and captured with a camera (DXC-950 P, SONY). Typically the magnification was 5.

Since the sections were larger than the usual specimens, they were imaged with SR micro-CT at a higher X-ray energy (28 keV) and in a specific mode allowing to virtually double the detector size. To this aim, the rotation axis of the specimen was shifted toward the side of the detector and the acquisition was performed over 360 degrees (with a double number of radiographs). Tomographic slices made of 4000×4000 pixels corresponding to a 5.6×5.6 mm region were finally reconstructed with a customized Filtered Back Projection reconstruction algorithm.

### 3D image analysis

A dedicated 3D automatic image analysis technique was developed to analyze microcracks. It involved two main steps: the segmentation of microcracks (i.e. their detection and the definition of their boundaries) and their quantification, respectively described in the two next sections. The entire images, each representing a volume of ∼30 mm^3^, were analyzed.

#### Segmentation of microcracks

Trabecular bone was initially segmented, which can be easily performed by simple thresholding due to the high image contrast. The trabecular bone envelope was then computed by using simple mathematical morphology to fill any voids [45]. The next step consisted in the segmentation of microcracks which was challenging due to the limited spatial resolution of images in comparison to the typical microcrack thickness and the level of noise in the image (cf. Fig. 1D). A pre-processing step removing ring artifacts and global intensity variations was first performed. Thus, a segmentation method taking advantage of the three-dimensional shape of microcracks was developed. Since microcracks are known to be planar defects, the contrast of planar structures in the 3D image was enhanced. To this aim, a 3D steerable filter based on the computation of the Hessian matrix at each voxel of the trabecular bone envelope was computed as described by Aguet et al. [46]. The response of this filter is high at the locations corresponding to planar patterns but can vary from an image to another, thus forbidding an automatic processing of series of images. Hence, this information was combined to a nonlinear filter inspired by the bilinear filter [47] to average gray-levels of voxels showing a similar planarity. This specific non linear filter allowed the enhancement of microcracks voxels by taking advantage of their local planarity (cf. Fig. 1D, middle). After this step, the resulting images were thresholded by hysteresis to extract microcracks and lacunae. Finally, each segmented object was labeled by performing a 3D connected component analysis [48] (Fig. 1D, right). The processing of all our images, required to tune a small number (<5) of parameters which were kept constant for the all images. In these conditions, we reckon that this method can be considered to be automatic.

The performance of the segmentation method was assessed on a large number of microcracks by an operator comparing the microcracks visible in the 2D original and segmented slices. By a careful observation of the overlay between the original image and the result of detection, such as in Fig 5, we could visually identify that only a few numbers of particularly thin microcracks were not detected by the automatic method, whereas the size and morphology of the segmented microcracks was in most cases well respected.

The 3D segmented microcracks were observed with the VGStudioMax software providing 3D renderings of voxel data sets.

#### 3D Quantification of microcracks

The quantification of microcracks and lacunae was performed from the labeled image. The volume, length, width, thickness of each labeled object **C,** was computed as follows. The microcrack volume (Cr.V) was obtained directly by counting the number of voxels.

Geometric moments are commonly used in image analysis, particularly for the shape analysis of binary objects. Let M_C_ be the second-order centered moment matrix of an object C :


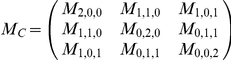
 with 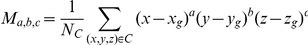


where N_C_ is the number of voxels of the component and 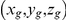 is its centre of mass. The diagonalisation of the moment matrix enables to find the main orientation of the structure through the eigenvectors. The eigenvalues are linked to the dimension of the structure in the three main directions, which are those of the ellipsoid having the same moment matrix. Explicitly, if 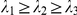 are the sorted eigenvalues of M_C_, the semi-axis lengths of the corresponding best-fitting ellipsoid, respectively denoted 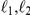 and 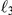
are given by 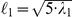, 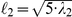
and 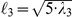.

This method is a conventional technique in 2D for providing the length and width of a structure of arbitrary shape. In the literature, the modelling of microcracks by flat ellipsoid has been considered [25].

The microcrack length (Cr.Le) and width (Cr.W), were respectively estimated by 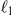 and 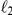. Due to possible twisting in the microcrack shape, 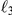 was not found to be a relevant estimator of thickness. The microcrack thickness (Cr.Th) was then estimated as the mean 3D direct thickness map defined at each voxel, as the diameter of the maximum sphere that can be included in the object [49]. Microcrack surface area (Cr.S) was evaluated as microcrack volume divided by thickness Cr.Th.

Microcracks and lacunae were discriminated according to their volume and thickness/width ratio. Porosities with a volume greater than 500 µm^3^ and a ratio inferior of 1/3 where identified as microcracks. Microcrack density Cr.Dn and lacunae density (La.Dn) were respectively evaluated as the number of microcracks and lacunae found in a specimen, divided by the trabecular bone volume (BV). Note that these densities are expressed in mm^−3^, and that since the number of microcracks was counted in three-dimensional space, a microcrack was counted only once, even when it was present on many different slices.

The trabecular bone volume fraction (BV/TV) and the Structure Model Index (SMI) of the whole specimen were also calculated from the segmentation of the trabecular bone envelope. For the selected microcracks, the thickness of the surrounding trabecula computed at the crack location (Tb.Th.lo) was evaluated at the site of the microcrack using the direct thickness map of the trabecular bone envelope. The trabecular shape, as a rod, plate or junction at the microcrack location, was computed automatically by analysing the local topology of the trabeculae [50].

### 2D Simulated quantification of microcracks

To compare these new 3D characteristics with published 2D data, 2D measurements were also computed from the 3D images. On that purpose, eight 7 µm-thick virtual sections of each specimen were created by averaging 5 consecutive SR micro-CT slices every 180 µm (128 slices). The 2D bone envelop of each section was identified with the same method as in 3D. The 2D bone volume fraction (BV/TV 2D) was measured by counting the number of voxels. Microcracks appearing in one of the eight sections were counted and measured manually. It was then possible to obtain the 2D number of microcracks (Cr.N 2D), the 2D microcrack density (Cr.Dn 2D), and their 2D mean microcrack length (Cr.Le 2D mean) as well as the standard deviation of these parameters.

## Supporting Information

Movie S1 3D animation of the six selected microcracks : linear (L2,L1,L3) (first line), parallel (P) (bottom left), cross-hatch (CH) (bottom center), union of twisted (X) (bottom right).
(AVI)
